# Complementary medicine in nursing homes - results of a mixed methods pilot study

**DOI:** 10.1186/1472-6882-14-443

**Published:** 2014-11-12

**Authors:** Miriam Ortiz, Eva Soom Ammann, Corina Salis Gross, Katharina Schnabel, Torsten Walbaum, Sylvia Binting, Herbert Felix Fischer, Michael Teut, Jan Kottner, Ralf Suhr, Benno Brinkhaus

**Affiliations:** Institute for Social Medicine, Epidemiology and Health Economics, Charité – Universitätsmedizin Berlin, Luisenstrasse 57, 10117 Berlin, Germany; Institute of Social Anthropology, University of Bern, Laenggasstrasse 49 a, 3012 Bern, Switzerland; Clinical Research Center for Hair and Skin Science, Department for Dermatology and Allergy, Charité - Universitätsmedizin Berlin, Charitéplatz 1, 10117 Berlin, Germany; Center for Quality in Care, Reinhardtstraße 45, 10117 Berlin, Germany

**Keywords:** Hydrotherapy, Kneipp, Complementary and alternative medicine, Elderly care, Nursing homes, Mixed methods research

## Abstract

**Background:**

‘Kneipp Therapy’ (KT) is a form of Complementary and Alternative Medicine (CAM) that includes a combination of hydrotherapy, herbal medicine, mind-body medicine, physical activities, and healthy eating. Since 2007, some nursing homes for older adults in Germany began to integrate CAM in the form of KT in care. The study investigated how KT is used in daily routine care and explored the health status of residents and caregivers involved in KT.

**Methods:**

We performed a cross-sectional pilot study with a mixed methods approach that collected both quantitative and qualitative data in four German nursing homes in 2011. Assessments in the quantitative component included the Quality of Life in Dementia (QUALIDEM), the Short Form 12 Health Survey (SF-12), the Barthel-Index for residents and the Work Ability Index (WAI) and SF-12 for caregivers. The qualitative component addressed the residents’ and caregivers’ subjectively experienced changes after integration of KT. It was conceptualized as an ethnographic rapid appraisal by conducting participant observation and semi-structured interviews in two of the four nursing homes.

**Results:**

The quantitative component included 64 residents (53 female, 83.2 ± 8.1 years (mean and SD)) and 29 caregivers (all female, 42.0 ± 11.7 years). Residents were multimorbid (8 ± 3 diagnoses), and activities of daily living were restricted (Barthel-Index 60.6 ± 24.4). The caregivers’ results indicated good work ability (WAI 37.4 ± 5.1), health related quality of life was superior to the German sample (SF-12 physical CSS 49.2 ± 8.0; mental CSS 54.1 ± 6.6). Among both caregivers and residents, 89% considered KT to be positive for well-being.

The qualitative analysis showed that caregivers perceived emotional and functional benefits from more content and calmer residents, a larger variety in basic care practices, and a more self-determined scope of action. Residents reported gains in attention and caring, and recognition of their lay knowledge.

**Conclusion:**

Residents showed typical characteristics of nursing home inhabitants. Caregivers demonstrated good work ability. Both reported to have benefits from KT. The results provide a good basis for future projects, e.g. controlled studies to evaluate the effects of CAM in nursing homes.

## Background

Current demographic changes in ageing societies are a major challenge for health care systems as well as for the social communities in all industrialised countries. An increasing number of care-dependent disabled older adults demands new concepts in preventive medicine, long-term treatment, and hospital care [1, 2]. In 2011, about 2.5 million individuals in Germany were care-dependent, and approximately 30% of them lived in nursing homes [3]. Chronic cardiovascular, musculoskeletal and metabolic diseases are common. Dementia is one of the most commonly diagnosed diseases in care-dependent older adults living in nursing homes: nearly two thirds suffer from it [4]. Therefore, a focus on prevention and maintenance of functioning levels is urgently needed to maintain quality of life (QoL) and to reduce morbidity in the elderly population. Experts in the field of Complementary and Alternative Medicine (CAM) suggest that CAM might offer preventive potential for senior citizens [5].

In 2007, some nursing homes for the elderly in Germany started to integrate CAM in the form of Kneipp Therapy (KT) in the daily basic care of their clients. KT is a form of prevention and treatment in the field of CAM and represents an important part of traditional European medicine, especially in the German-speaking parts of Europe. CAM KT methods are well known in the German population [6]. They can be traced back to the European medicine traditions and were formulated mainly by Sebastian Kneipp, a Catholic priest and a non-professional medical practitioner living in the 19^th^ century. He developed a large range of self-help and therapeutic strategies including hydrotherapeutic interventions, herbal medicine, mind-body medicine, physical activity, and healthy nutrition [7]. In Germany, the approximately 160,000 member Kneipp Association keeps this CAM tradition alive and provides professional education in KT.

‘Kneipp nursing homes’ implement those interventions in daily care and are mostly differentiated from conventional nursing homes through offers referring to hydrotherapy and herbs. For minor ailments, often simple herbal teas or aromatherapy are offered by the nursing staff. Many ‘Kneipp nursing homes’ maintain herb beds and organic vegetable gardens. In the field of hydrotherapy, various applications are offered by trained personnel such as wraps, layers, foot or arm baths, treading water and dry brush. Nutrition in Kneipp nursing homes relies on healthy, fresh, seasonal, whole food with a high proportion of fruits and vegetables. Elements of mind-body medicine range from relaxation to creative therapy offers. Physical activity is offered in groups (e.g. gymnastics class, garden walks) or as individualized physiotherapy or occupational therapy. The idea of KT is mostly to regulate or stimulate body and mind functioning via frequent mild stimuli, e.g. from hydrotherapy or physical activity but also from mind-body elements. The intention is to improve physical functions and quality of life, taking into account the well-being of the individual.

To achieve certification as a ‘Kneipp nursing home’, the management must provide a concept of integration of KT in daily routine care, which has to be validated by the Kneipp Association. At least three persons on the staff have to be trained in KT by the Academy of the Kneipp Association. Kneipp trainers are, together with the nursing home management, responsible for implementing KT in the nursing homes’ daily living and care routines. To date there are 18 ‘Kneipp nursing homes’ in Germany.

The aim of this research project was to gather information about the integration of KT in daily routine care in four Kneipp nursing homes, and to report on the health status of the residents and caregivers who received respectively applied KT. In addition, after the implementation of KT, changes subjectively experienced by residents and caregivers were investigated in the qualitative research component.

One underlying aim of this study was to use the findings as a basis to generate adequate research questions, identify feasible and relevant assessment tools, and gather experience in terms of feasibility for conducting a further study on the effects of KT.

### Study design

This research project was performed as a cross-sectional, mixed methods study including a quantitative (part 1) and a qualitative (part 2) component in a convergent parallel design [8]. It was conducted between September and December 2011 in four certified Kneipp nursing homes in two German states (Bavaria (n = 2) and North Rhine-Westphalia (n = 2)). The study was performed in accordance with the Declaration of Helsinki and was approved by the ethics commission at the Charité - Universitätsmedizin Berlin (EA1/147/11; 22th June of 2011). Trial registration: DRKS00006800 (25th September of 2014).

## Methods - part 1: quantitative component

### Nursing homes

At the time this study began, there were four certified Kneipp nursing homes in Germany. All of them could be recruited for our study. Nursing home A was located in a rural area in Bavaria, and had at the time of study entry 136 residents and 117 employees. Nursing home B was located in North Rhine-Westphalia in the center of a city and had 74 residents and 87 employees. Nursing home C was located in a small town. At the time of the study it had 63 residents; 70 persons were employed. Nursing home D was located in a rural area of Bavaria and had 44 seniors and 35 employees. Every nursing home provided outside and inside facilities for Kneipp hydrotherapy, medicinal herb beds, space for exercise and relaxation therapy, and in-house kitchens for meal preparation for the residents. KT was offered regularly by parts of the caregiver teams or therapists.

With the help of the respective Directors of Nursing, we conducted a pre-screening of the residents on the basis of the main in- and exclusion criteria. On the basis of this screening, we were able to contact legal guardians for residents under guardianship and inform them about our study before we initiated interviews. Caregivers and residents (and, if necessary, legal guardians) were informed verbally as well as in written form about the study content. Caregivers and residents who provided written informed consent and fulfilled inclusion criteria were included in the study. Assessments for residents were performed by specially trained and experienced study personnel. Caregivers received questionnaires by letter. All assessments and questionnaires were documented in case report forms for each study participant.

### Study population

Inclusion criteria for residents were an age of at least 60 years, the ability to answer questions adequately, written and oral informed consent (for those under legal guardianship, guardians had to provide consent) and regular (daily or weekly) individualised KT for at least 3 months. Inclusion criteria for caregivers were an age of at least 18 years, regular and routine delivery of KT in the nursing home for at least 3 months, and at least 3 years general professional experience.

### Assessments

The activities of daily living (ADLs) were measured with the Barthel-Index. This questionnaire is a recommended assessment and often used in healthcare to refer to daily self-care activities as a measurement of the functional status of a person [9]. ADLs include feeding oneself, bathing, dressing, grooming and the ability to move; the Barthel Index scores ADLs on a scale from 0 to 100 (0 = very dependent, 100 = not dependent) [10, 11]. The Quality of Life in Dementia (QUALIDEM) is a dementia-specific QoL instrument, which was developed for use in residential care. We used the version for people with mild to severe dementia which consists of 37 items, divided in 9 subscales regarding care relationship, restless tense behavior, positive affect, negative affect, positive self-image, social relations, having something to do, feeling at home, and social isolation. It is rated by professional caregivers or proxies. Results can be described as points or percents of the scale for each item [12]. The Profile of Well-being is a tool that reflects the well-being of residents. Caregivers evaluate residents’ well-being subjectively within 14 indicators regarding signs of positive affect, communication, creativity, activity, cooperation, humour, and self-respect [13]. The Short Form 12 Health Survey (SF-12) describes the health-related QoL including physical and mental health aspects [14–16]. To assess cognition, we performed the Mini Mental Status Examination (MMSE), which is a 30-point test measuring arithmetic, orientation, and memory functions [11, 17, 18]. In addition, the residents were asked about use, knowledge, meaning, preferences, and the perception of KT regarding their well-being. Demographic and further variables like care level (it defines the grade of care dependency from grade I to III), diagnoses, medication were taken from the nursing records. Predetermined questions about KT were asked of the residents in a standardized way, and the Mini Mental Status Examination was carried out face-to-face between residents and the study staff. All other assessments were external assessments and performed with the help of the respective caregivers who had to reflect on the situation of their clients to answer the questionnaires.

The following variables were assessed in caregivers: The Work Ability Index (WAI) Short Form evaluates work ability and comprises 10 questions including aspects of physical and psychological work demands, health status, and reserve capacity. The WAI yields a continuous score ranging from 7 to 49 points, where higher scores indicate better work ability. WAI scores can be categorized as excellent (44–49 points), good (37–43 points), moderate (28–36 points) or poor (7–27 points) [19–21]. To evaluate overall health-related QoL we used the SF-12 self-evaluation form [14–16]. In addition, caregivers were asked how long they have been familiar with KT, if they use KT for their own health issues, what kind of KT they deliver and how often, and their preference for particular forms of KT for self-treatment and for the treatment of residents. Additionally, caregivers were asked if KT is supposed to have effects or not for their own health or the health of residents, if and how KT changes the relationship between caregiver and resident, and how KT can be integrated in usual care in terms of feasibility. All caregivers received questionnaires by letter and returned them to the study secretary.

### Data management and statistical analyses

Data management was conducted according to ICH-GCP guidelines. All data for residents and caregivers were analysed descriptively with R Development Core Team (Vs. R 2.14 [22]) and SAS (Vs. 9.2). Results for continuous data were reported as means and standard deviations or medians, and for nominal data as absolute or relative frequencies.

## Results - part 1: quantitative component

### Residents

**Figure 1:**
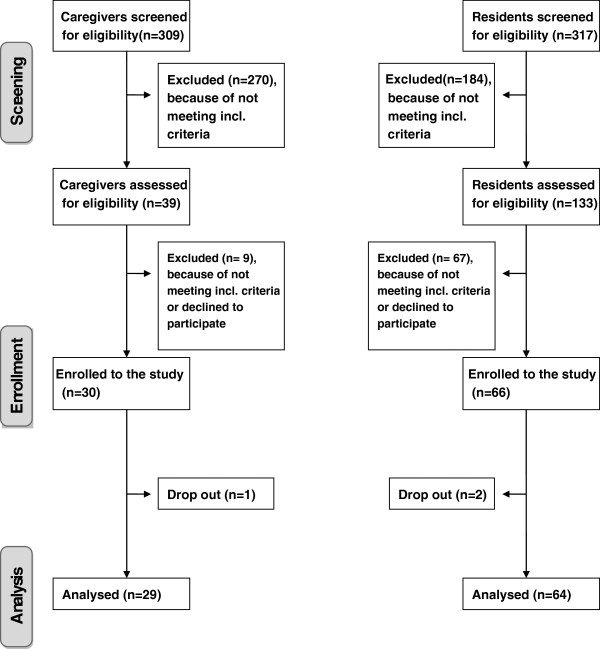
**Study participants´ flow chart.**

The pre-screening on the basis of the main in- and exclusion criteria identified 133 out of 317 residents (the total of all residents of the four nursing homes) as eligible for inclusion in the study. In a second screening step we identified again 46 residents not fulfilling the inclusion criteria, 16 residents declined to participate, one died, one was at the hospital and three legal guardians could not be contacted. In the end, 66 residents were included. Two residents dropped out, thus 64 residents were considered for the analyses (Figure 1 Study participants´ flow chart). More than two thirds (83%) of the assessed residents were female with a mean age of 83.2 (SD ±8.1) years (Table 1). The number of diagnoses ranged between 3 and 14 with a mean of 8 (SD ±2.9) diagnoses per resident. The diagnoses documented most frequently were hypertension (56%), musculosceletal diseases (51%), metabolic diseases such as diabetes (31%), coronary heart disease (25%), dementia (42%) and depression (25%). Residents took on average 8 (SD ±3.0) different drugs daily, mainly for cardiovascular diseases (38%), gastrointestinal diseases (14%), for psychiatric disturbances (12%) and for pain (8%). Residents in our study were distributed along a care continuum (as defined by the German Social Code Book XI) ranging from 6% at no care level, 55% at care level 1, 33% at care level 2, and 6% at care level 3.

**Table 1:** **Socio-demographic data of residents and caregivers (quantitative component)**

	n	Gender female	Age (years)*	Height (cm)*	Weight (kg)*	BMI (kg/m ^2^)*
**Residents**	64	n = 53 (82.8%)	83.2 ± 8.1	161.9 ± 9.3	72.1 ± 16.1	27.4 ± 5.4
**Caregivers**	29	n = 29 (100%)	42.0 ± 11.7	166.7 ± 6.2	76.3 ± 16.6	27.3 ± 5.9

BMI = Body Mass Index, SD = Standard Deviation, n = Number, *Mean ± SD.

The mean of the Barthel Index was 60.8 points (SD ±24.4) (13% had a Barthel Index between 0 and 30 (severe disability), 64% between 35 and 80 (moderate disability), and 23% more than 85 points (nearly no disability). The cognition test (the MMSE) resulted in an average of 22.3 points (SD ±6.3) (29% between 0 and 18 points (severe to moderate cognitive impairment), 29% between 19 and 24 (mild cognitive impairment) and 42% more than 25 points (no cognitive impairment). The results of the SF-12 showed an average of 43.2 (SD ±8.1) for the physical component summary scale and 56.9 (SD ±8.2) for the mental component summary scale. The highest ratings on the QUALIDEM subscales were gathered for ‘feeling familiar’ (91%), ‘social isolation’ (89%) and ‘positive affect’ (88%) (high ratings for ‘social isolation’ means less marked). The Profile of Well-being showed an average of 25.2 points (SD ±3.1) (Table 2). All residents received each of the different elements of KT once or twice a week. When asked about what they think KT consists of, residents primarily associated KT with hydrotherapy (88%), followed by herbal treatments (53%) and physical activity (45%). Among residents, 43% were aware of KT since adulthood; 26% KT since childhood; 23% since their move into the Kneipp nursing home, 8% could not answer the question. Among residents, 71% preferred hydrotherapy as their primary KT intervention. The majority of residents (89%) perceived KT as positive for well-being.

**Table 2:** **Outcome parameter of residents (quantitative component)**

	n	Mean (±SD)	Scale range (points)
**Barthel-Index**	64	60.8 ± 24.4	0-100
**MMSE**	52	22.3 ± 6.3	0-30
**QUALIDEM**			
Nursing relationship	64	18.5 ± 3.5 (88%)	0 - 21
Positive affect	64	15.9 ± 3.0 (88%)	0 - 18
Negative affect*	64	7.2 ± 1.7 (80%)	0 - 9
Restless, tense behaviour*	64	5.3 ± 1.4 (58%)	0 - 9
Positive self-perception	64	7.1 ± 2.3 (78%)	0 - 9
Social relationships	64	14.1 ± 3.8 (78%)	0 -18
Social isolation*	64	8.0 ± 1.6 (89%)	0 - 9
Feeling familiar	64	11.0 ± 2.2 (91%)	0 - 12
Having something to do	64	3.4 ± 2.0 (56%)	0 - 6
**Profile of Well-being**	64	25.2 ± 3.1	0-28
**SF-12** Physical Comp. Sum. Scale	64	43.2 ± 8.1	0-100
**SF-12** Mental Comp. Sum. Scale	64	56.9 ± 8.2	0-100

MMSE = Mini Mental Status Examination, SF = Short Form, SD = Standard Deviation, n = Number.

*higher rating means less marked.

### Caregivers

The pre-screening identified 39 caregivers out of a group of 309 staff members (drawn from all professional fields in the nursing home) as eligible for study participation because they regularly applied KT to residents (Figure 1). Nine caregivers could not be included because they were not available (n = 4) at the time of evaluation, refused study participation (n = 2), or did not respond (n = 3). Thirty caregivers were included in the study, but one did not return the assessment forms. In the end, the data provided by 29 caregivers was analysed. All caregivers were female and on average, 42 years old (SD ±11.7) (Table 1). Caregivers had worked an average of 10 years in their professions, 55% full-time, 41% part-time, and two-thirds worked as shift workers.

The Work Ability Index of the caregivers showed an average of 37.4 (SD ± 5.1) points, reflecting a ‘good’ work ability. The SF-12 of the caregivers showed an average of 49.2 (SD ± 8.0) for the physical component summary scale and 54.1 (SD ± 6.6) for the mental component summary scale (Table 3). When starting their work in the Kneipp nursing home, 48% of the caregivers first came into contact with KT; 93% used KT for themselves, mainly in the of hydrotherapy or physical activity. Among caregivers, 96% reported subjective positive effects of KT on their wellbeing and health. Caregivers preferred hydrotherapy (65%) and mind-body methods (44%) for resident care (multiple answers could be given). The majority of the caregivers (90%) stated that their relationship to the residents had improved since implementing KT. About 47% stated an improved relationship to the caregiver team as a result of KT and 42% stated that KT could be easily integrated into their daily work.

**Table 3:** **Outcome parameters of caregivers (quantitative component)**

	n	Mean (± SD)	Scale range
**SF-12** Physical Comp. Sum. Scale	28	49.2 ± 8.0	0-100
**SF-12** Mental Comp. Sum. Scale	28	54.1 ± 6.6	0-100
**Work Ability Index**	23	37.4 ± 5.1	7-49

SD = Standard Deviation, n = Number.

## Methods - part 2: qualitative component

The qualitative study component aimed at describing everyday KT practice in nursing homes and residents’ and caregivers’ subjectively perceived changes following the implementation of KT. We approached this aim by doing a rapid appraisal based on ethnographic fieldwork techniques (including participant observation and semi-structured interviews) in two of the above-mentioned nursing homes. Both nursing homes were similar in size, resident population and organizational structure, one located in a small town (nursing home C) and one in a rural area (nursing home D). During a one-week observation period in each of the two nursing homes, residents and caregivers were accompanied by an ethnographically trained researcher (ESA, a social anthropologist experienced in researching health and care organizations but not in CAM or KT) who observed them in their daily KT activities from early morning to the evening. During this one-week observation period, semi-structured interviews focusing on subjective experiences of change were conducted with selected residents and caregivers, as well as with KT trainers, heads of nursing and directors of the two nursing homes. Participants were selected by theoretical sampling among those caregivers and residents being present during the one-week field stay. In addition, directors, heads of nursing and the KT trainers responsible for KT implementation were systematically included. In total, 26 interviews were conducted. Participant observation and interviewing focused on pre-defined aspects of subjective perspectives (Table 4), which were transformed into practice-oriented open questions and collected in a field manual. The questions were mainly directed at generating narrative accounts on experiences with KT. Analysis of the interview transcripts and field notes were adapted to the explorative character of the study design and loosely followed the principles of Grounded Theory (open, axial and selective coding by the researcher who did the fieldwork) [23, 24].

**Table 4:** **Aspects of subjective perspectives of residents and caregivers (qualitative component)**

Residents	Caregivers
• Experience of care and naturopathic applications	• Experience of care and naturopathic applications
• Therapeutic relationship	• Relationship with residents
• Health complaints	• Professional self-concept
• Illness experience	• Illness perceptions and concepts
• Illness perceptions and concepts	• Working conditions, job satisfaction
• Self-efficacy, control of reinforcement, sense of coherence	• Stress
• Perspectives on the future	• Identification with the employing organization
	• Motivation
	• Quality of care, caring competencies
	• Co-operation within the caring team
	• Self-experiences with naturopathy

Note: These items were pre-defined and informed a set of practice-oriented open questions collected in a field manual. Questions were situationally adapted to meet the interviewee (be it nursing home directors, heads of nursing, nurses, nurses’ aides or residents).

## Results - part 2: qualitative component

Both nursing homes included in the qualitative component of this study showed an integral implementation of KT principles, including individual care, group therapies, social activities, nutrition, and a specific arrangement of spaces allowing for spontaneous Kneipp activities. Implementing KT in this kind of holistic approach is in accordance with the certification requirements of the German Kneipp Association, which, as the directors and heads of nursing stated, is associated with an intense reflection on how the nursing home organizes care and daily activities and with what aims (see Table 5, second box on ‘conceptual focus’). KT is integrated into daily activities directed at all residents, such as healthy menu planning, collective meals, social gatherings, moderate physical activities (e.g., going for a walk in fresh air, group activities), and also offered as individual treatment. Kneipp activities and treatments are thus in one or another form available to every resident – and, to a certain extent, also to the caregivers – in the nursing home. Residents and staff most commonly associated KT with individually applied forms of hydrotherapy such as washing, baths, gushes, and massages.

**Table 5:** **Systematic overview of interpretive categories re implementation (qualitative component)**

Nursing home C	Nursing home D
**Type‚ specialized implementation‘**	**Type‚ integrative implementation‘**
Individual KT treatments conducted by a KT trainer (→ specialized knowledge)	individual KT treatments conducted by all nurses and nurses’ aides (→ generalized knowledge)
**Conceptual focus on attentive dimensions:**	**Conceptual focus on physical-sensual dimensions:**
*‘I think that Kneipp is a conception sensitizing us for things we already do in elder care. To let us have a closer look on how we do things in care and what effect we want to achieve. For example the right nutrition, or being there for someone. Yes, it’s a holistic view on care. Everyone is talking about holistic care, but this is a hazy expression, what can you do with it? And I think that the Kneipp concept is describing what holism is.’ (head of nursing)*	*‘I do think that Kneipp is giving the whole thing a name, or a roof. A bit of orientation, so that the staff knows what is important to us, and the residents know it as well, their relatives, everyone knows that we have a slightly different way of working here, another kind of consciousness about care.’ (director)*
*‘I think what makes a difference is that care is done in a conscious manner. There are a lot of things one already does in care, but it is not done consciously, although it is at the same time a Kneipp treatment. It’s about the attention given in that moment, by the nurses. For example at lunch, when they feed someone, if you do it with ease, take a chair and sit next to the resident instead of standing and pushing the spoon in – this would also be a treatment in the Kneipp way, feeding with consciousness and ease and giving attention through it.’ (KT trainer)*	*‘Maybe it works so well because it’s so normal. I mean, I could just as well work with any kind of sound therapy or scents or whatever, but that’s rather special. Kneipp, instead, is down-to-earth, I do not have to explain it to the residents, they know it and they understand it.’ (director)*
*‘Well, it is simply part of our profession that we work here under a high tension, that we do not always have the inner calmness necessary to transfer our attention to the resident. For example, if we do not feel comfortable and calm ourselves, we could do Kneipp ten times and it would not reach the residents. No, it would only become hectic and have no effect for the resident.’ (a nurse)*	*‘Simply as far as skin care is concerned, or decubitus prophylaxis, Kneipp treatments are just the optimal thing. Washing with cold water and brushing the skin is but perfect, better than all those ridiculously expensive skin products we used in other nursing homes to enhance the blood circulation of the skin, we do not need those things here! We do very simple things that don’t cost anything.’ (head of nursing)*
	*‘I think that Kneipp makes a difference about care because we have slightly more time for the residents. For example when we brush the skin, you need to take your time to brush every part of the hand or the arm, and with the washrag you always to it tatata and done. If you use the brush, it’s a little more time you give. And, after all, it’s not the same thing every day! One day you brush, one day you wash with cold water, one day you prepare a bath. And we would all get fed up with having to eat spinach and eggs every day, don’t we? And it’s the same with basic care.’ (a nurse)*
**Holism: the entire organization is ‘doing Kneipp’**	
Explanation of symbolic order: director, head of nursing and KT trainer	explanation of symbolic order: director and head of nursing
Keepers of specialized knowledge: KT trainer and a few nurses/nurses’ aides externally trained in KT	keeper of specialized knowledge: head of nursing (who is a trained KT trainer)
Knowledge transfer: voluntary internal schooling by KT trainer	knowledge transfer: compulsory element of job introduction for nurses and nurses’ aides
Application of KT treatments: KT trainer (according to trainer’s treatment plan)	application of KT treatments: care staff (according to residents‘ treatment plans)
Additional KT activities: care staff (voluntary, within daily basic care activities); attendants (individual attendance in daily activities); therapists and social workers (their activities are integrated into the KT concept); kitchen crew (cooking healthy menus)	additional KT activities: nurses’ aides, attendants and volunteers (group activities and individual attendance in daily activities); therapists (their activities are integrated into the KT concept)
**Personalized application, complex treatments**	**Pragmatic application, simple treatments**
KT treatments are done by the KT trainer, in a manner that stresses individual attention (giving time, serving the individual needs of the resident)	Head of nursing instructs the staff how to apply KT
Therapist applies complex, time-consuming treatments, which are popular among the residents (hot/cold baths, massages, hot rolls etc.)	Each staff member applies KT according to pragmatic instructions
Nurses and nurses’ aides are invited to apply KT as well, but do it seldom because they do not feel in a position to give the same amount of time and individual attention as the KT trainer does	Treatments are chosen that integrate well into the daily tasks and routines of care (washings, gushes, brushing, simple baths etc.)
A few nurses and nurses’ aides punctually apply single elements in basic care (e.g. brush massages) and in treatment of indispositions (e.g. herbal teas, poultices)	Residents get a fixed treatment plan compulsory for staff
**Application of KT in the mode of a gift**	**Application of KT in the mode of a standard service**
No time pressure: KT treatments can be done in a careful, individually adapted manner and therefore stress the attentive aspects. Only the KT trainer does treatments; frequency and regularity is hard to achieve.	KT treatments are done regularly, several times a week. This requests planning, offers liability for residents, and obliges staff to apply KT.
Treatments have an enchanted character; they are individual gifts of absolute attention.	Treatments have a pragmatic, everyday character; they are part of the standard services.
Treatments focus on well-being and indulging.	Focus on simplicity (cold washes, gushes) and regularity also leads to observable physiological effects; therefore, residents and staff tend to be convinced about positive long-term effects on health.
Treatments and the person of the therapist are very popular among residents.	‘Cold‘ treatments are regarded as unpopular among residents, which leads some team members to replace unpopular treatments by more appreciated ones (such as the brush massages); this brings in the gift dimension (cf. organization C).
Nurses and nurses’ aides acknowledge that ‘doing Kneipp’ is ‘something beautiful‘ they do not have the possibilities to do in their daily care work.	
**Residents’ agency: non-negotiable, gratitude**	**Residents’ agency: negotiable, a right**
Residents may co-determine KT within the concrete interactions during a treatment since treatments focus on situational needs of the residents.	Residents have a therapy plan in their rooms and know what treatments they are supposed to get. Treatments are therefore part of standard services the residents have a right to.
Treatments are closely tied to the person of the therapist and tend to be experienced as personal and comprehensive ‘caring about’.	Residents may claim treatments on the basis of this plan, they may also negotiate situational changes in treatments (e.g. receiving a brush massage instead of a cold washing). They may, however, not influence who does the treatment (i.e. KT is not person-bound).
Residents have no explicit claim to receive treatments; they are perceived as occasional gifts, not regular services.	The power to define KT lies with the head of nursing (who puts up the treatment plan); the power to apply KT lies with the staff, but is negotiable for the residents.
The power to define and to apply KT treatments is not perceived to be available to residents.	
**Outcome for the residents: gain in attention and well-being**	
*‘Sometimes they treat you here as if you were a piece of wood. And Ms. X (the KT trainer) is always very kind. One day she makes me a hot roll, another day a hot-cold foot bath. And I somehow feel better afterwards.’ (a resident*	*‘When I came here and saw those pictures of Mr. Kneipp hanging everywhere – we had them at home as well when I was a child! Yes, Kneipp was always present at our home, and certainly this helped me get so old. Just today I had one of those cold washings – freezing it was, I thought I am not going to survive it! But now I feel so well, so warm.’ (a resident)*
*‘It always feels good. It’s good if you get an opportunity to relax, one feels less stiff, I can move better, blood circulation is better, this does a lot. And I like Ms. X (the KT trainer), her entire personality is good.’ (a resident)*	*‘Well, the dry brushing, this is great, really. It releases, and it wonderfully stimulates blood circulation, and it feels very well. I am always looking forward to this!’ (a resident)*
	*‘Yes, one is grateful for that, if it itches at your back, if someone washes or brushes you there. And one can have such nice talks with the nurses while they’re doing it.’ (a resident)*
**Outcomes for the organization: uniqueness and secondary gains from more contented residents**	
Gains for organization: uniqueness, i.e. the Kneipp nursing home is a better place to reside and a better place to work; more continuity in staff; lower material costs (medication, skin care products)	
Gains for staff: emotional and functional gains from more contented residents; wider scope of action (especially nurses’ aides), more variety in basic care	
Limitations: time; compulsion to ‘do Kneipp’	
*‘Since Kneipp is so multifaceted there are so many possibilities to apply something, small but sometimes powerful. Be it with teas for example, doing small things with big effects.’ (a nurses‘ aide)*	*‘I am in a position to offer something to the residents, so that they feel like: Now they’re doing something special for me.” (a nurses’ aide)*
*‘Take for example a fever: before you grab the paracetamol, you can try to do a calf packing, which is not a big thing.’ (a nurse)*	*‘Well, to be honest, a contented resident also uses his bell less often.’ (a nurse)*
*‘And if you do some Kneipp and see how much joy they get from what you do for them, then (laughs) you want to have more of that!’ (a nurse)*	*‘If someone is contented, if I was able to help him or her with small things, then this helps me as well. I can stay with other work, I am more contented as well, everyone is happier!’ (a nurses’ aide)*
*‘When Mrs. W. gets her depressions, for example, she does not call us when she needs to go to the toilet. And when she feels well – and Kneipp is good for her psyche – she also cooperates better in care.’ (a nurse)*	*‘If you see reactions from residents you did not expect, it’s joyful, it’s nice, somehow. That’s the kick in nursing the elderly, it makes you happy if you get reactions, and if you get appreciation for what you do.’ (a nurse)*

The qualitative component identified two different types of KT implementation showing effects on how KT is perceived (Table 5): Type 1 is characterized by a specialized implementation: The nursing home employs a KT trainer, who is responsible for applying KT in addition to the conventional care activities of caregivers. In this implementation type, treatments are perceived by the residents as an exceptional care activity, applied with the intention to foster their individual well-being. This leads to a resident‘s perception of KT as a personal gift (i.e. a transaction focusing on long-term reciprocity [25]) and thereby promoting an exclusive relationship between the resident and the KT trainer. Type 2 is an implementation type focused on including KT in basic care, where treatments are delivered by all the caregivers. This implementation type gives rise to KT being perceived as an everyday service (i.e. a commodity) and tends to de-personalise the way KT is experienced (each person working in care is capable of delivering it, while in Type 1 the experiences associated with KT are closely related to the individual person of the KT therapist). On the other hand, type 2 empowered residents to actively request KT since it was perceived as part of the standard service to which each resident has equal access. As this brief characterization of the observed implementation types shows, there are diverse implementation possibilities for KT in nursing home care, but each has crucial consequences for the experiences of residents and caregivers and for the ways in which KT is perceived.

The two observed types of KT implementation in institutional elder care also differ in their focus either on personalised attention or physiological aspects of KT: Treatments as individual gifts tend to emphasize attention, while treatments perceived as everyday commodities allow for regular applications promising better effects on the body. However, both types of implementation have been perceived by the residents and the caregivers as fostering a substantially more attentive and more individualised culture of caring. ‘*We are now explicitly allowed to give attention, to sit next to the bed and hold a hand*’, a nurses’ aide has put it. The interviews with residents furthermore clearly showed that residents receiving individual KT treatments experienced them as unique and personal (see also Table 5). Moreover, KT is described by the residents and the caregivers as being compatible with the lay knowledge of the residents and with their perceptions of what is good for their health and well-being. The fact that the nursing home is trying to do something good to their health and well-being by using KT is therefore tangible and understandable for the residents. Some of the residents also stated that they were aware of their own possibilities “to do Kneipp” and live healthy. However, the fact that nursing home residents are of advanced age, live with a severely restricted health and must rely on care from other persons clearly restricted their sense of agency and self-determination.

Although some caregivers state that the integration of KT results in a slightly increased expenditure of time in basic care activities, others also observe time gains resulting from the less time-consuming behavior of more content and quieter residents. The directors and heads of nursing of both participating nursing homes stated that integration of KT was possible without increasing the personal or financial resources needed for care.

The analysis of the data collected in participant observation and interviews showed that the integration of KT generated benefits in three respects: for the nursing home itself, for the caregivers and the residents (see also Table 5): First, as the responsible actors (directors, heads of nursing) stated, the nursing home as an organization enjoys the benefit of leveraging KT as a marketing tool, distinguishing the Kneipp nursing home from other homes. From an organizational perspective, KT is perceived to offer the security of a frame of reference for all actors involved. Furthermore, there is the potential for more content and possibly healthier residents, as well as cost savings with regard to medication and personal care products. Besides that, the planning and conceptualization of KT integration is a highly appreciated opportunity for in-depth organizational self-reflection since KT implementation is not simply about adding treatments. Second, residents potentially experience the following benefits: As stated by both caregivers and residents, there is a clear gain in attention and contentedness for the residents, especially for those receiving regular individualized KT. Furthermore, residents experience more variety and individuality in care (e.g. when washing in the morning is done in different ways on specific days, according to an individual weekly treatment plan). Since KT uses treatment elements which are widespread in local folk medicine, residents also state a feeling of acceptance of their lay knowledge. Third, caregivers mainly report experiencing emotional and functional gains through more contented residents. Furthermore, caregivers appreciate the larger variety in caring procedures. Due to this and due to the possibilities of KT to ease discomfort in many ways, caregivers also state that they experience a widened scope of action through the integration of KT. Furthermore, KT offers a legitimization for attentive aspects of caring since giving attention is a fundamental element of good care according to KT and not a potential waste of time.

Limitations that were mentioned first include the expenditure of time by management and staff to implement KT in the organization. Second, residents referred to a restricted sense of control since they are in constant need of care. Third, as some caregivers stated, the integration of KT, due to its holistic dimensions affecting all dimensions of working in a nursing home, may also be experienced as a normative compulsion by some team members.

## Discussion

To our knowledge, this is the first study that evaluated residents and caregivers in nursing homes working with KT. Considering the overall lack of caregivers in elder care in Germany and the rising demographic of aged persons the perspective of caregivers who call for a different, multi-dimensional and more self-determined routine care is an especially promising aspect. To generate further research questions and to gain as much and as complex information as possible about residents and caregivers, in this study we combined a quantitative with a qualitative approach. This mix of methods allows eclectic insight into the research topic from a more generalizable viewpoint (quantitative) as well as from the perspective of the involved individuals (qualitative). Due to space limitations, it is not possible to report every detail of the different study components, however, publications are planned to address additional detail of the individual components.

A limitation of the quantitative component was that the inclusion criteria limited the study sample to a small group of residents and caregivers. We did not expect that only a relatively small number of the caregivers applied individualized KT. Although there were elements of KT (e.g. nutrition, group activities) applied to all residents, only some of the residents received individual KT treatments (e.g. hydrotherapy) regularly. Thus the results cannot be generalized to other caregivers and residents of the nursing homes. A further limitation is the external rating for most of the residents’ assessments. Data might be biased due to varying qualifications of the raters. In addition, more recent studies show that the QoL of the raters may also influence external ratings [26].

The qualitative component of our project was focussed on an exploratory appraisal of how KT integration is experienced by residents and caregivers in two nursing homes. It might be possible that further qualitative research would reveal additional integration types with distinct effects on experiences and perceptions of residents and caregivers.

Finally, the design of this project does not allow conclusions about any effects at all of integrating KT. Indeed some of the interviewed caregivers stated potential benefits in the qualitative component of the study, which allowed us to develop new research questions and outcomes for future studies, but it is of course not possible to generalize those individual statements.

The results derived from both components of the study demonstrate that it seems possible to integrate KT in the daily routine of the nursing homes although residents were clearly restricted. Furthermore, the acceptance of KT, and especially for hydrotherapy, was high and considered to be beneficial for well-being by most of the study participants. In addition, the caregivers demonstrated a good work ability and quality of life. They appreciated KT both in applying it to the residents and using it for themselves. Favored treatments for self-care among caregivers were hydrotherapy and exercise. Among caregivers, 90% stated an improved relationship to their clients because of the changes perceived since the integration of KT.

In terms of age, gender, multi-morbidity and poly-pharmacy, the sample of the quantitative component was comparable to the overall German nursing home population [27, 28]. Activities of Daily Living (Barthel Index) demonstrated clear restrictions [10]. Although restricted activities of daily living often have a negative impact on QoL, we found relatively good results for the QoL assessments. The QUALIDEM scores for the subscales ‘feeling familiar’, ‘social isolation’, ‘care relationship’, and ‘positive affect’ were rated high in comparison to other studies [29]. These results are consistent with the results of the qualitative component reporting subjectively perceived gains in attention and well-being for the residents.

The ‘Profile of Well-being’ is a rarely used multidimensional instrument for evaluating QoL by a caregiving team. Compared to residents in shared housing arrangements, well-being scores were high [30]. Also the results for health-related QoL measured by the SF-12 were on average superior to the German sample >70 years (physical component summary scale and 38.8 (SD ±10.6), mental component summary scale 52.3 (SD ± 9.2)) [14]. But it has to be stated critically that there are no comparable data for an externally evaluated SF-12, so this may also have an influence on the distinctive results for the mental sum scale. QoL might be related to several determinants such as depression, neuropsychiatric symptoms (e.g. irritability, anxiety, and aggressiveness), psychiatric drug use and restricted activities of daily living [31–33]. While the role of cognition is discussed, this may have influenced our results for QoL because the results for the MMSE reflected only moderately impaired cognition; 42% even had MMSE scores >25. Maybe the inclusion of residents who were ‘able to answer questions adequately’ influenced the results for the MMSE. Nearly 70% of the residents knew KT before they moved to the nursing home, which may had an influence on evaluating it to be beneficial for well-being. However, the residents interviewed in the course of the qualitative component all stated that KT was not the main reason to choose the nursing home.

The results for the 29 participating caregivers indicated on average a ‘good’ work ability (WAI) in the sample comparable to other German nursing homes and health care settings [34, 35], while the health-related QoL represented by the SF-12 was superior to the German sample for healthy women for both the mental and physical component summary scale [14, 19]. A great majority of caregivers used elements from KT for their own health and well-being, which shows the possible impact of KT for primary or secondary prevention as well as for overall health awareness.

The results of the qualitative component showed that the integration of KT in nursing homes did not simply add a therapeutic element, but tended to change the culture of care in the nursing homes in general [36] (see also Table 5), shifting the focus from professionalism, efficiency and quality measures to a holistic perspective stressing attention, sensitivity and well-being. Integrating the Kneipp naturopathy concept in a long-term care facility seems to be associated with intense reflections on how care can become compatible with the central principles of KT. Integration therefore fosters changes, not only by adding hydrotherapeutic treatments and herbal medicine, but also in promoting moderate physical activity, healthy eating, and elements structuring the social lives and mental balance of residents. Although different types of KT implementation have been observed, having different effects on how KT is perceived, the cultures of care in Kneipp nursing homes seem to contribute to a ‘holistic conception’ of care that can be traced back to the early 1960s nursing theorists [37]. This also involves an explicit legitimacy of the attentive and emotional aspects of caring, such as giving time, respecting individual moods and preferences, and having fun, as well as enjoying attention and tactile care, possibly without increasing the personal or financial resources needed for care. As a recent systematic review of qualitative studies has shown, attentive caring and an explicit focus on relationship-centered approaches to care seem to be of considerable importance for residents’ well-being in nursing homes [38]. Furthermore, KT relates to well-known traditional concepts of folk medicine, which were reported by both residents and caregivers to convey a sense of acceptance of the lay knowledge and the life experiences of the residents. KT seems to be a well-understood therapeutic concept working with simple and everyday means. Therefore, KT has a certain potential to foster residents’ interactive health literacy and co-determination, although only within the restricted scope of action of individuals in need of care.

Although both residents and caregivers stated that KT primarily produces benefits for the residents, there are also indirect gains for the caregivers, as has been reported. Contented residents not only contribute to lighter workloads, but their well-being and the gratitude that often is expressed after a Kneipp treatment is also perceived as positive feedback and appreciation for the caring personnel. With their focus on personal attention and their legitimation for attentive aspects of care, Kneipp nursing homes practice a relationship-centered approach, which has been well established as having an important role in dealing with future challenges in long-term care [1, 38–40]. In sum, the subjectively perceived changes induced by KT implementation in nursing homes point to a concept with the potential to develop new cultures of care focusing on the residents’ well-being and on their health promotion – an orientation that appears to hold promise in coping with the present and future challenges in long-term care [1, 38].

For further studies it might be interesting to find out if benefits, including increased care and attention paid to the residents, as well as a reduction of residents’ complaints, may not only satisfy residents but also lead to higher job satisfaction among caregivers and improve the subjective conceptualization of caregivers’ roles [41, 42]. Therefore, the integration of CAM interventions in routine care may lead to an increasing job diversity and differentiation, thus making work in nursing homes attractive to more people [43, 44]. Due to the shortage of caregivers in Germany, particularly in nursing homes for older adults, this could be advantageous.

## Conclusion

The results of this study including quantitative as well as qualitative research components suggest that the integration of KT in nursing homes is accompanied by a high acceptance among the involved residents and caregivers. Caregivers demonstrated a good work ability and health related QoL. Residents suffered from a restricted health status. Both residents and caregivers reported that KT was perceived as positive on residents’ well-being and on the attention they received in care. Results provide a sufficient basis for future research projects including controlled studies to evaluate the effects of KT in nursing homes.
